# Delineation of autoantibody repertoire through differential proteogenomics in hepatitis C virus-induced cryoglobulinemia

**DOI:** 10.1038/srep29532

**Published:** 2016-07-12

**Authors:** Masato Ogishi, Hiroshi Yotsuyanagi, Kyoji Moriya, Kazuhiko Koike

**Affiliations:** 1 Department of Infectious Diseases, Graduate School of Medicine, The University of Tokyo, Tokyo, Japan; 2 Department of Infectious Control and Prevention, Graduate School of Medicine, The University of Tokyo, Tokyo, Japan; 3 Department of Gastroenterology, Graduate School of Medicine, The University of Tokyo, Tokyo, Japan

## Abstract

Antibodies cross-reactive to pathogens and autoantigens are considered pivotal in both infection control and accompanying autoimmunity. However, the pathogenic roles of autoantibodies largely remain elusive without *a priori* knowledge of disease-specific autoantigens. Here, through a novel quantitative proteogenomics approach, we demonstrated a successful identification of immunoglobulin variable heavy chain (V_H_) sequences highly enriched in pathological immune complex from clinical specimens obtained from a patient with hepatitis C virus-induced cryoglobulinemia (HCV-CG). Reconstructed single-domain antibodies were reactive to both HCV antigens and potentially liver-derived human proteins. Moreover, over the course of antiviral therapy, a substantial “de-evolution” of a distinct sub-repertoire was discovered, to which proteomically identified cryoprecipitation-prone autoantibodies belonged. This sub-repertoire was characterized by IGHJ6*03-derived, long, hydrophobic complementarity determining region (CDR-H3). This study provides a proof-of-concept of *de novo* mining of autoantibodies and corresponding autoantigen candidates in a disease-specific context in human, thus facilitating future reverse-translational research for the discovery of novel biomarkers and the development of antigen-specific immunotherapy against various autoantibody-related disorders.

Chronic infections of viruses such as hepatitis C virus (HCV) and human immunodeficiency virus (HIV) can frequently be accompanied by immune complex disease and autoimmunity^1^,^2^. Broadly neutralizing antibodies (bNAbs) against such viruses are often polyreactive and/or autoreactive, representing a double-edged nature of humoral immunity in infection control and deterioration of self-tolerance^3^. However, it remains unclarified whether circulating antibodies with the highest affinity and neutralization potential against pathogens have the highest importance in infection-driven autoimmunity. The lack of *a priori* knowledge of etiological autoantigens hinders affinity purification-based approaches. In view of this dilemma, we hypothesized that autoantibodies of the highest etiological significance could be defined by the following criteria: (i) highly enriched in a disease-specific compartment *in vivo*; (ii) reactive to antigens of the causative pathogen and self-antigens; and (iii) diminishing after eradication of the causative pathogen. In this work, we developed a methodological framework termed “differential proteogenomics”, and demonstrated a successful delineation of autoantibody sub-repertoire and corresponding autoantigen candidates by analyzing clinical specimens isolated from an HCV-infected female patient with mixed cryoglobulinemia (referred to as UT1). UT1 achieved a sustained virologic response after a six-month treatment with daclatasvir (DCV) and asunaprevir (ASV), which also ameliorated her dermatologic and arthritic symptoms. Detailed clinical information is summarized in Supplementary Table 1. Integrated analysis of quantitative proteogenomics and longitudinal antibody repertoire profiling revealed the most disease-specific autoantibody sub-repertoire and corresponding autoantigen candidates. The methodological framework presented here can be applied to a variety of autoantibody-related disorders to screen disease-specific autoantigens and autoantibodies, paving the way for the discovery of novel biomarkers and the development of antigen-specific immunotherapy.

## Results

### Antibodies highly enriched in cryoprecipitate were proteogenomically identified

**Figure 1:**
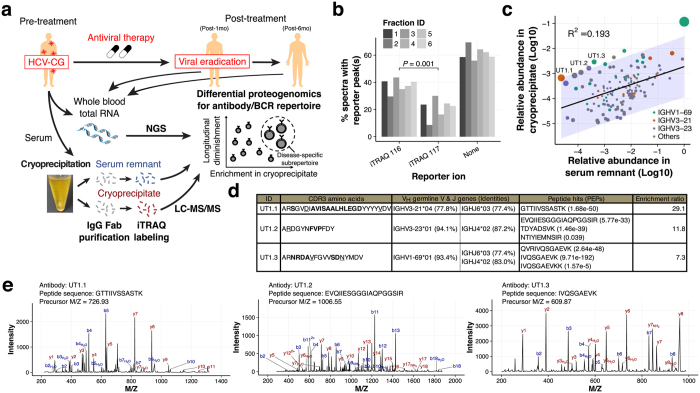
Identification of V_H_ sequences highly enriched in cryoprecipitate. (**a**) Schematic illustration of experimental framework. HCV-CG, hepatitis C virus-induced cryoglobulinemia. Illustrations were modified from the resources distributed in the Togo picture gallery (http://g86.dbcls.jp/~togoriv/), licensed under CC-BY 4.0 ©Togo picture gallery by the Database Center for Life Science (DBCLS), Japan. (**b**) Labeling efficiencies of iTRAQ reporter tags. Fab samples from cryoprecipitate and serum remnant were labeled with iTRAQ reporter reagents 116 and 117, respectively. The labeling efficiency was defined as the ratio of the number of spectra containing the iTRAQ peak of interest to the total number of acquired spectra. Statistical comparison was carried out through the Welch’s *t*-test. (**c**) Correlation plot of relative abundances of identified V_H_ sequences. Generally, a weak correlation was observed between the abundance of each V_H_ sequence in serum remnant and that in cryoprecipitate. The size of each dot represents Cook’s distance, which indicates how outlying that sequence is. The band represents a 95% prediction interval. (**d**) Summary table for three representative V_H_ sequences selected on the basis of their relative enrichment in cryoprecipitate. For CDR-H3 amino acid sequence, bold indicates non-templated regions, and underline indicates somatic mutations. PEP, posterior error probability. (**e**) Representative mass spectra of the evidence peptides of three selected V_H_ sequences: left, UT1.1, middle, UT1.2, right, UT1.3.

The study outline is illustrated in Fig. 1a. We first isolated Fab fragments from resolubilized cryoprecipitate and serum remnant. The isolated Fab fragments were labeled with the isobaric tag for relative and absolute quantification (iTRAQ)^4^ for quantitative comparison. The labeling efficiencies differed between samples as expected owing to the unknown difference in amino acid composition (38 and 21% for tag 116 and 117, respectively; *P* = 0.001) (Fig. 1b). We normalized the raw iTRAQ reporter intensities by an equimolar spike-in BSA to offset the effect of different labeling efficiencies. Simultaneously, we constructed a personal IgG V_H_ database as previously reported^5^,^6^,^7^. To minimize false-positive hits due to excessive redundancy and sequencing errors in reference database, we adopted the unique molecular identifier (UMI) strategy^8^. Rarefaction analysis indicated a sufficient sequence depth for identifying unique UMI groups (Supplementary Fig. 1). The database constituting 3,605 unique V_H_ sequences was searched using MaxQuant^9^ with a peptide and protein false discovery rate (FDR) threshold of 0.01. Peptide-spectra matches (PSMs) with only one or no iTRAQ peak were discarded, since they have smaller differences between the top and second-highest PSM scores and higher average mass deviations, indicating low-confidence matching due to the intrinsically high similarities of V_H_ sequences^7^ (Supplementary Fig. 2). Eventually, 150 unique V_H_ sequences were identified (Fig. 1c). We focused on three IGHV gene segments, IGHV1-69, IGHV3-21, and IGHV3-23, on the basis of previous reports that these gene segments are frequently utilized in the context of chronic infections and autoimmune diseases including HCV-CG^10^,^11^,^12^. Three sequences (designated as UT1.1, UT1.2, and UT1.3) were selected as representatives (Fig. 1d,e and Supplementary Fig. 3). Most of the evidence peptides for UT1.1-1.3 were unique to the UT1 database, not being discovered in the control database (Supplementary Fig. 2).

### Proteogenomically isolated antibodies were reactive to HCV and self-antigens

**Figure 2:**
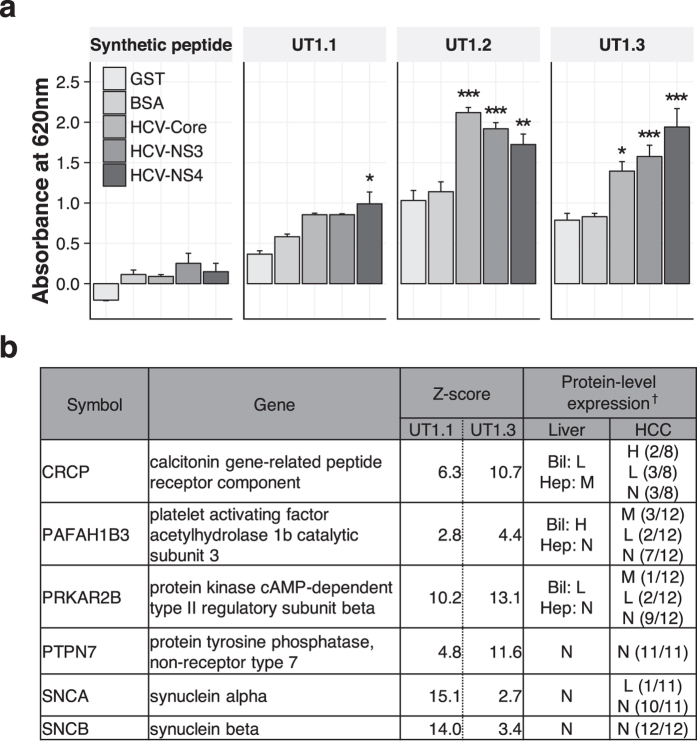
Reactivity profiles of representative V_H_ sequences. (**a**) Reactivity against HCV core and nonstructural (NS) antigens was measured via indirect ELISA. Background-subtracted absorbance values are shown. The synthetic peptide has the same sequence to the C-terminus of single-domain antibodies studied. Since two-way ANOVA revealed significant reciprocal relationships, multiple groups were compared by pairwise one-way ANOVA followed by Tukey post hoc test, using the pairw.anova function implemented in the asbio package in R. Adjusted *P* values against GST control are presented according to the following codes: *P < 0.05, **P < 0.01, ***P < 0.001. Bars indicate mean ± s.e.m. N = 3 per group. Experiments were performed in duplicate and repeated twice. (**b**) Summary table of autoreactivity profiles identified in the HuProt protein array experiments. No autoreactivity was identified for UT1.2. Completely overlapping candidate autoantigens were identified for both UT1.1 and UT1.3 when candidates with Z-score of 2.5 or less were ignored. ^†^Protein-level expression data were retrieved from The Human Protein Atlas^16^. Bil, bile duct cells. Hep, hepatocytes. HCC, hepatocellular carcinoma. The codes H, M, L, and N denote high, medium, low, and no detectable expression, respectively.

The selected V_H_ sequences were expressed as single-domain antibodies, the minimum form that keeps their original specificities^13^ (Supplementary Fig. 4). Core and NS3 antigens are considered particularly important in cryoprecipitate formation in HCV-CG^14^,^15^. Significant reactivity to core and NS3 antigens was revealed for UT1.2 and UT1.3, whereas UT1.1 showed only borderline reactivity to NS4 antigen (Fig. 2a). Next, we profiled their autoreactivity profiles using a human proteome array (Fig. 2b). Interestingly, UT1.1 and UT1.3 showed completely overlapping autoreactivity profiles against self-antigens, for some of which expressions have been observed in the liver and hepatocellular carcinoma (HCC)^16^. On the contrary, UT1.2, derived from IGHV3-23 gene segment, was not autoreactive as previously reported^12^. Taken together, UT1.1, UT1.2, and UT1.3 appeared to be a bystander autoantibody, an anti-HCV antibody, and a cross-reactive autoantibody, respectively.

### CDR-H3 amino acid hydrophobicity was associated with cryoprecipitation

**Figure 3:**
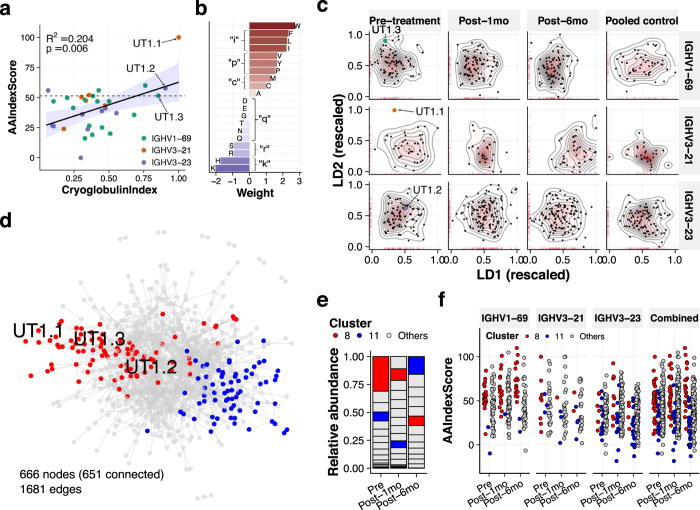
Longitudinal dynamics of CDR-H3 repertoire architecture over antiviral therapy. (**a**) Construction of AAIndexScore as the best predictive axis for CryoglobulinIndex, which is defined as a rescaled ratio of iTRAQ signal intensities. A dash line represents the best threshold value determined by receiver operating characteristic (ROC) analysis. (**b**) Relative contributions of amino acid residues to AAIndexScore. Lowercase alphabetical characters indicate amino acid categories classified through a hierarchical clustering approach. (**c**) Similarity-based two-dimensional mapping of CDR-H3 repertoires. On the basis of the amino acid categories shown in (**b**), edit distance between each CDR-H3 sequence pair was calculated. The resultant distance matrix was converted to coordinates through a principal coordinate analysis (PCoA) followed by a linear discriminant analysis (LDA). Longitudinal shifts were particularly notable in IGHV1-69 and IGHV3-21 repertoires, but not in IGHV3-23 repertoire. (**d**) Similarity network analysis of CDR-H3 repertoires. IGHV1-69, IGHV3-21 and IGHV3-23-derived CDR-H3 sequences in different time points were combined to construct a single network, in which each CDR-H3 node was connected to their most-similar counterparts on the basis of the distance defined in (**c**). Representative clustering results obtained by multilevel optimization algorism is presented. Red, Cluster8. Blue, Cluster11. Interestingly, CDR-H3 sequences of UT1.1-1.3 were classified into the same cluster. (**e**) Longitudinal shrinkage of Cluster8 identified in (**d**). (**f**) Distributions of AAIndexScore. CDR-H3 sequences in Cluster8 generally have higher AAIndexScore than those in Cluster11. Meanwhile, no notable time-dependent changes in AAIndexScore were observed.

Chronic HCV infection is a good model of infection-driven autoimmunity in that antiviral treatment enables elimination of causative pathogen. As anticipated, transient increase in the selection pressure in CDR accompanied by the increase in overall CDR-H3 clonotype diversity was observed after antiviral therapy (Supplementary Fig. 5). To further dissect the differences between longitudinal V_H_ repertoires in UT1, we explored the best axis to parametrize the propensity for condensation to cryoprecipitate, defined as a rescaled ratio of iTRAQ intensities (referred to as “CryoglobulinIndex” hereafter). We hypothesized that CryoglobulinIndex is largely determined by the biochemical properties of CDR-H3 region. Since CDR-H3 parameters such as length, charges, and GRAVY (grand average of hydropathy)^17^ do not necessarily reflect the disease specificity of HCV-CG, we pursued a bottom-up approach; we selected a set of amino acid variables that best distinguish highly enriched CDR-H3 sequences from non-enriched ones. The best combination consisted of hydrophobicity parameters (Supplementary Fig. 6 and Supplementary Table 3). This regression score was designated as “AAIndexScore” (Fig. 3a,b). Higher weights were assigned to hydrophobic residues including V, L, I, and M, consistent with previous studies showing hydrophobic interactions of these residues with HCV antigens^18^.

### Autoantibody repertoire delineated via amino-acid-level similarity network analysis shrunk after HCV eradication by antiviral therapy

Amino acids were classified into eight categories based on their relative weights in AAIndexScore (Fig. 3b). Using these categories, we defined amino-acid-level distances between CDR-H3 pairs. Principal coordinate analysis and linear discriminant analysis revealed a longitudinal shift of CDR-H3 repertoires, most notably in IGHV3-21 repertoire (Fig. 3c). To identify the most dynamically affected sub-repertoire, we next adopted a network clustering approach. Interestingly, UT1.1-1.3 sequences were classified in the same cluster (Cluster8) by multilevel optimization algorism (Fig. 3d). Multiple algorisms generated similar results (data not shown). Cluster8 shrunk longitudinally, whereas Cluster11, positioned at the opposite of Cluster8 in the similarity network, expanded after six months (Fig. 3d,e). Meanwhile, no qualitative differences were observed between time points in terms of AAIndexScore, CDR-H3 length, and GRAVY score (Fig. 3f and Supplementary Fig. 7). Consistent with the relative weights in AAIndexScore, negatively charged CDR-H3 sequences were abundant in Cluster8 before antiviral therapy (Fig. 3b and Supplementary Fig. 7).

**Figure 4:**
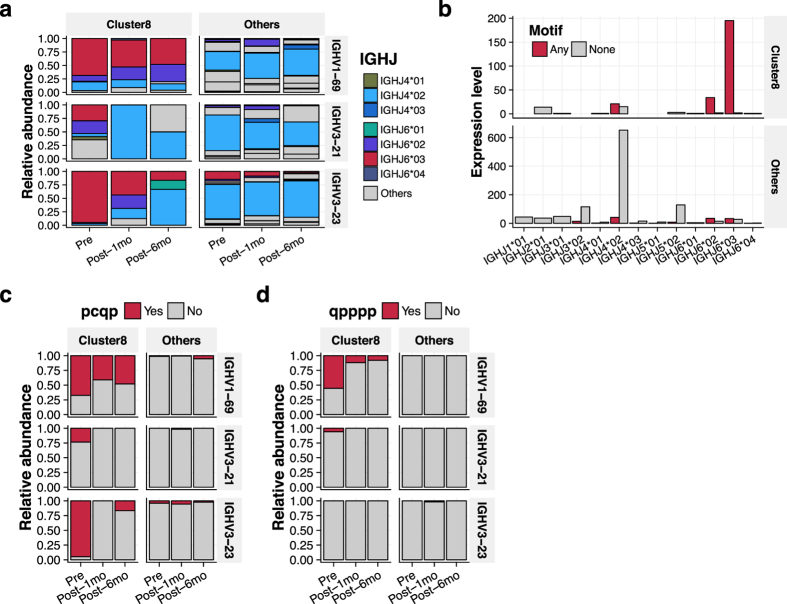
Feature characterization of the most dynamically shrinking CDR-H3 sub-repertoire. (**a**) Cluster8 was enriched with CDR-H3 sequences derived from IGHJ6. Longitudinal shrinkage of sub-repertoire derived from IGHJ6, particularly IGHJ6*03, was observed. (**b**) Motif screening. CDR-H3 amino acid sequence motifs were screened by univariate analysis. Note that amino acids categorized as defined in Fig. 3b were used for the motif analysis. Nineteen motifs were retained after filtering by the threshold of Bonferroni-adjusted *P* value < 0.05. Most of the motifs significantly enriched in Cluster8 were of IGHJ6*03 origin. (**c,d**) Diminishing trends of two representative motifs, “pcqp” and “qpppp”. In most cases, “pcqp” and “qpppp” correspond to IGHJ6-derived sequences YMDV and D/EYYYY, respectively. Of note, UT1.1 contains “qpppp”, and UT1.3 contains “pcqp”.

IGHJ6 gene segments, most notably IGHJ6*03, were enriched in Cluster8 (Fig. 4a). The relative abundance of IGHJ6*03-derived sequences decreased after antiviral treatment, whereas in the pooled control almost no IGHJ6*03-derived sequences were identified (Supplementary Fig. 8). A univariate analysis identified 19 motifs enriched in Cluster8, most of which were derived from IGHJ6*03 and to less extent IGHJ6*02 (Fig. 4b). Interestingly, UT1.1 contains “qpppp”, a motif with the highest odds ratio, and UT1.3 contains “pcqp”, a motif with the smallest *P* value (Fig. 1d). Both motifs appeared to be derived from IGHJ6*03, and decreased after antiviral treatment (Fig. 4c,d).

### Publicly available anti-HCV and anti-HIV neutralizing antibodies (NAbs) showed similar CDR-H3 amino acid compositions

To explore whether the AAIndexScore and accompanying findings in this study has some generalizable implications in a broader context, we investigated the AAIndexScore distributions on several publicly available human antibody sequences^19^,^20^,^21^,^22^. Intriguingly, publicly available anti-HCV and anti-HIV NAbs, especially those with IGHJ6-associated motifs, share significantly higher AAIndexScore, indicating that our AAIndexScore may reflect some generalizable features of autoreactivity-prone virus-neutralizing antibodies elicited in chronic infections (Supplementary Fig. 9 and Supplementary Tables 4–8).

## Discussions

Our study validates the methodology of delineating disease-specific autoantibody sub-repertoire only using clinical isolates in human patients. Due to the profound difference of the immune systems of experimental animal models and humans, etiologies of many possibly immune-related disease entities remain elusive. Thus, unsupervised identification of autoantibody sub-repertoire is an attractive attempt that may help deepen our insights, establish clinically useful immune biomarkers, and pave the way toward antigen-specific immunotherapy^23^.

Chronic infections, particularly of viral origins, have been suspected of triggering and/or aggravating autoimmune diseases^1^,^2^,^24^,^25^. Of note, virus-neutralizing antibodies with IGHJ6-derived long CDR-H3 tend to originate from inherently self-reactive immature B cell populations^17^,^26^,^27^,^28^. The poly-Y residues encoded by IGHJ6 genes are critical to the neutralization of HIV in some bNAbs^29^. Consistently, a rapid shrinkage of the IGHJ6-rich sub-repertoire after viral eradication observed in this study may reflect virus-driven expansion of the cross-reactive B cell population in the context of HCV-CG (Supplementary Fig. 5 and Figs 3 and 4). On the other hand, positively charged residues contributed to lower AAIndexScore (Fig. 3b), whereas previous studies have associated positive net charge with self-reactivity^17^,^28^. This discrepancy might be explained by the strong conservation of positively charged residues in hypervariable region 1 of the HCV envelope E2 protein^30^, possibly indicating the advantage of negatively charged and/or hydrophobic residues for anti-HCV antibodies.

Our key hypothesis is that antibodies enriched in the most disease-specific compartments *in vivo* should be of the highest etiological importance. Consistently, three representative antibodies highly enriched in cryoprecipitate showed reactivities against HCV antigens and overlapping cross-reactivities against human proteins whose expression in the liver has been reported^16^ (Fig. 2). Notably, the weak anti-HCV reactivity of UT1.1, the antibody most highly enriched in cryoprecipitate, illustrates the limitation of the affinity-oriented strategy. Meanwhile, UT1.3 shows cross-reactivity against HCV antigens and several autoantigens. PAFAH1B3 is, inter alia, of particular interest, since this protein possibly triggers global lipidomic alteration and oncogenesis in relation to various oncogene pathways^31^,^32^. The upregulation of PAFAH1B3 in HCV-bound hepatocytes and in HCV-induced HCC has also been reported^33^,^34^. Possibly, B cells cross-reacting with HCV and self-antigens discharged from hepatocyte preferentially proliferate in the liver microenvironment to serve as a protective immunity against both chronic hepatitis C (CHC) and ongoing oncogenic processes, given the observation that HCV-CG patients show a lower incidence of HCC than those without CG^35^. Concurrently, the universal expression of PAFAH1B3, including the thyroid and salivary glands, may explain some of the extrahepatic manifestations of CHC (Supplementary Fig. 10).

This study is a proof-of-concept attempt, thus having several limitations. First of all, this is a single-patient report. The generalizability of the findings should be validated in a larger cohort. Another limitation is that this work is observational, and should not be interpreted as a definitive evidence for the causal responsibility of cryoprecipitate-constituting autoantibodies cross-reactive to both HCV and autoantigens including PAFAH1B3 in the autoimmune aspect of HCV-induced cryoglobulinemia. Moreover, the effect of differences in direct-acting antiviral regimen on antibody repertoire dynamics should further be explored, as pharmaceutical substances themselves might also trigger some host immune responses. Nonetheless, the consistency of our results with previous studies suggests the plausibility of our strategy of differential proteogenomics, warranting further research. We believe our attempt is a first and valuable step toward elucidation of the etiologies of various autoantibody-related disorders in a real-world setting.

In summary, our study demonstrated the feasibility of delineating autoantibody repertoire and autoantigen candidates *de novo* in a disease-specific context. Considering the double-edged nature of antibodies, unveiling the origin and pathological implications of infection-driven autoantibodies could be a milestone for the discovery of novel biomarkers and the development of effective therapeutic strategies against both chronic infections and autoantibody-mediated systemic disorders.

## Methods

### A patient

The study participant (UT1) was a 70 year-old female patient with chronic hepatitis C and clinical symptoms of cryoglobulinemia. Her HCV genotype was 1b. She had a dermatologic and arthritic symptoms aggravated with low temperature. She was positive for cryoglobulin. As serum monoclonal protein was not proven via immunoelectrophoresis, her cryoglobulinemia was likely to be type III. She was treated with a combined regimen of daclatasvir (DCV) and asunaprevir (ASV), achieving SVR12. Her clinical data over the course of antiviral treatment are summarized in Supplementary Fig. 1. She provided written informed consent. Treatment with direct-acting antivirals and subsequent follow-up examinations were carried out at our clinic in The University of Tokyo Hospital. This study protocol conformed to, and was conducted in accordance with, the ethical guidelines of the Declaration of Helsinki. This study was approved by the Research Ethics Committee of the University of Tokyo (permission number: G10078).

### Computational analysis

All computational analyses were conducted using R ver. 3.2.3 (https://www.r-project.org/)^36^ and/or Wolfram Mathematica ver. 10.2 (https://www.wolfram.com/mathematica/)^37^. The latest versions of R packages were consistently used. Scripts are available upon request.

### Isolation of cryoprecipitate

Cryoprecipitation was conducted as previously described^38^. Five milliliters of a whole-blood specimen was collected into a vial without an anticoagulant. The specimen was allowed to clot in an incubator at 37 °C. After clotting and sedimentation, approximately 4 mL of the supernatant was collected into new tubes, and refrigerated at 4 °C for 7 days. After centrifugation, serum remnant was collected into a new vial. The resultant cryoprecipitate was washed with binding buffer (20 mM sodium phosphate, pH 7.0), and resolubilized in 100 μL of binding buffer containing 1% sodium dodecyl sulfate.

### Quantitative LC-MS/MS analysis

The resolubilized cryoprecipitate and serum remnant were diluted with binding buffer up to 10 mL. Each sample was mixed with 200 μL of prewashed Protein G Mag Sepharose (GE Healthcare, Little Chalfont, UK) and incubated at room temperature for 1 h. Beads were collected using a magnet plate, washed twice with binding buffer, and eluted three times with 100 μL of elution buffer (0.1 M glycine-HCl, pH 2.7) at room temperature for 5 min. The three eluates were immediately neutralized with 10 μL of neutralization buffer (1 M Tris-HCl, pH 9.0), and combined. IgG was fragmented and Fab was purified using Pierce Fab Micro Preparation kit (Thermo Fisher Scientific Inc, MA, USA), strictly following the manufacturer’s instructions. The Fab fragments collected were stored at 4 °C until subsequent analysis.

Protein samples were precipitated with trichloroacetic acid (TCA), resolubilized in resolubilization buffer (50 mM triethyl ammonium bicarbonate (TEAB) containing 0.1% SDS, pH 8.5), and quantified by ultraviolet spectroscopy. Protein was then spiked with 1 μg of BSA as an internal control, reduced, cysteine-blocked, and digested with trypsin at 37 °C for 20 h. Digested peptides were labeled for 3 h at room temperature using iTRAQ reagents in accordance with the instructions included in the iTRAQ Reagents Multiplex kit (Applied Biosystems, CA, USA). Peptides derived from cryoprecipitate were labeled with the iTRAQ 116 reporter, and peptides from serum remnant were labeled with the iTRAQ 117 reporter. After labeling reaction, proteins were purified by cation-exchange chromatography, desalted, fractionated into six fractions using Agilent 3100 OFFGEL Fractionator (Agilent Technologies, CA, USA). Each fraction was desalted, and examined by LC-MS/MS. Mass spectra were acquired using a Thermo Scientific LTQ Orbitrap XL mass spectrometer (Thermo Fisher Scientific).

### V_H_ repertoire sequencing

Nine milliliters of a whole-blood specimen was drawn into three Tempus Blood RNA tubes (Life Technologies, CA, USA). The tubes were immediately shaken vigorously to lyse cellular components and stabilize RNA, and stored at −20 °C until use. RNA was purified using a Tempus Spin RNA Isolation kit (Life Technologies) according to the manufacturer’s instructions. The quality and quantity of purified RNA were measured using NanoDrop 3300 fluorospectrometer (Thermo Fisher Scientific). RNA was ethanol-precipitated and stored at −20 °C as a pellet in ethanol.Total RNA from peripheral leukocytes isolated from pooled Asian donors was purchased as a control (Clontech, CA, USA).

All primers were synthesized by Life Technologies. Template-switching oligo (TSO) containing the unique molecular identifier (UMI) barcode sequence and locked nucleic acid (LNA) at the 3′ terminus^39^ was synthesized by Exiqon. Oligonucleotide sequences used in this study are summarized in Supplementary Table 2. Reverse transcription (RT) was performed as follows. The RNA mixture consisted of 1 μL of 1 μg/μL of template RNA, 1 μL of 1 μM gene-specific RT primer, and 1 μL of 10 mM dNTP mixture (Life Technologies). After preheating at 72 °C for 3 min, the RNA mixture was mixed with 7 μL of the RT mixture consisting of 1.5 μL of RNase-free distilled water (DW), 2 μL of 5 x SMARTScribe First-Strand buffer (Clontech), 1 μL of 20 mM DTT (Clontech), 1 μL of 1 μM TSO, 0.5 μL of 20 U/μL SUPERase-In RNase inhibitor (Life Technologies), and 1 μL of 100 U/μL SMARTScribe reverse transcriptase (Clontech). The RT mixture was gently mixed by tapping, incubated at 42 °C for 60 min, and inactivated at 70 °C for 15 min. To amplify template-switching products efficiently, we adopted the step-out and suppression PCR technique^40^. The first PCR reaction mixture consisted of 3.6 μL of DW, 4 μL of 5 M Betaine (Sigma-Aldrich, MO, USA), 10 μL of 2 x KAPA2G Fast Multiplex PCR mix (Kapa Biosystems, MA, USA), 0.6 μL of 1 μM heel-carrier forward primer, 0.4 μL of 10 μM heel-specific forward primer, 0.4 μL of 10 μM reverse primer, and 1 μL of non-purified cDNA. The thermal cycling conditions were programmed as follows: denaturation at 95 °C for 3 min, 18 cycles of denaturation for 15 s at 95 °C, annealing for 30 s at 60 °C, and elongation for 30 s at 72 °C, followed by final elongation at 72 °C for 1 min. The second PCR reaction mixture consisted of 4.2 μL of DW, 4 μL of 5 M Betaine, 10 μL of 2× KAPA2G Fast Multiplex PCR mix, 0.4 μL of 10 μM forward primer, 0.4 μL of 10 μM reverse primer, and 1 μL of non-purified first PCR product diluted 1000-fold with DW. The thermal cycling conditions were the same as those of the first PCR, except that the total number of cycles was increased to 30. Twenty microliters of second-PCR products were directly applied to 2% agarose gel and electrophoresed. Single bands approximately 600 bp in length were visualized using ultraviolet light. DNA was purified from excised bands using MinElute Gel Extraction kit (Qiagen, CA, USA). Eluted DNA was repaired using PreCR Repair mix (New England Biolabs, MA, USA). The repair reaction mix consisted of 30.5 μL of DW, 10 μL of eluted DNA, 5 μL of 10× ThermoPol reaction buffer, 0.5 μL of 10 mM dNTP mixture (Life Technologies), 0.5 μL of β-nicotinamide adenine dinucleotide (NAD^+^), 2.5 μL of 20 mg/ml BSA, and 1 μL of PreCR Repair mix. The repair reaction mixture was incubated at 37 °C for 30 min, and re-purified using Agencourt AMPure XP kit (Beckman-Coulter, CA, USA). The repaired PCR products were eluted with TE Buffer, quantified using Qubit 3.0 Fluorometer (Life Technologies), and stored at 4 °C.

Paired-end libraries were prepared from 200 ng of DNA inputs using a TruSeq Nano DNA Sample Prep kit (Illumina, CA, USA), strictly following the manufacturer’s instructions. Adapter-ligated DNA fragments at a size distribution of approximately 800 bp were enriched using SPRI beads. Eight cycles of PCR were carried out using barcoded primers to enrich DNA inserts flanked with adapters. PCR products were purified, pooled, and 2 × 300 bp paired-end sequencing was performed using MiSeq and MiSeq Reagent kits V3 (Illumina).

### Identification of V_H_ sequences enriched in cryoprecipitate

UMI-guided de-multiplexing and quality filtering were performed using MiGEC^8^. Paired-end sequences were merged. UMI groups (MIGs) of less than five reads were discarded. VDJ gene segments were identified and aligned using IMGT/HighV-QUEST^41^. VDJ sequences were extracted to construct a personal immunoglobulin amino acid sequence library for MS/MS database search. Protein identification and quantification were performed using MaxQuant^9^. The intensities of iTRAQ reporters were normalized on the basis of equimolar spike-in BSA (1 μg per 100 μg sample). The relative degree of condensation to cryoprecipitate was defined as follows:


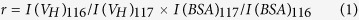


where *I*_116_ and *I*_117_ represent the intensities of the iTRAQ 116 and 117 reporters, respectively.

### Recombinant single-domain antibody (sdAb)

V_H_:V_L_ pairing information was inevitably lost during the mass spectrometry analysis. Therefore, we decided to focus on V_H_ throughout the study. Indeed, V_H_-derived single-domain antibody is likely to be sufficient for qualitatively determining the reactivity and specificity of the original antibody^13^.

Plasmids encoding the amino acid sequences of V_H_ sequences of interest were synthesized through the GeneArt service (Life Technologies). Codons were optimized for *Brevibacillus* species using GeneOptimizer and by manual inspection. As described previously, three mutations (G44E, L45R, and W47G) were introduced to improve the solubility of human IGHV when expressed as sdAb^42^,^43^. Recombinant sdAb was fused to a dual tag composed of 6xHis and EPEA amino acid sequence (C-tag) for subsequent purification and immunochemical probing^44^. The structure of the sdAb was: [V_H_ region including FR4]-EPKTPKPQHHHHHHGYQDYEPEA. Recombinant sdAbs were produced using the *Brevibacillus* expression system as previously described^45^. The culture conditions were as follows: for UT1.1, 100 mL of 2SY medium (TakaraBio, Shiga, Japan) without 0.2 M arginine at 30 °C for 48 h; for UT1.2 and UT1.3, 100 mL of 2SY medium plus 0.2 M arginine at 30 °C for 48 h. Bacteria were removed by centrifugation and subsequent filtration (0.8 μm). Secreted sdAbs were dialyzed twice with Dialysis Buffer (20 mM sodium phosphate and 0.5 M NaCl, pH 7.4). Purification was performed by using a nickel-sepharose column. Proteins were eluted by gradient elution using dialysis buffer and elution buffer (dialysis buffer plus 0.5 M imidazole, pH 7.4). The collected proteins were filtered, mixed with a Protein Stabilizing cocktail (Thermo Fisher Scientific), and stored at 4 °C until use.

### ELISA against HCV antigens

Assays were performed twice in duplicate. The antigens, antibodies and reagents used were as follows: HCV core genotype-1b, NS3 genotype-1b, and HCV NS4 mosaic antigen genotype-1 (ProSpec, NJ, USA); UltraPure BSA (Thermo Fisher Scientific); GST (Sigma-Aldrich); and CaptureSelect anti-C-tag biotin conjugate and Pierce High Sensitivity Streptavidin-HRP (Thermo Fisher Scientific). Antibodies were diluted with Western BLoT Immuno Booster reagent (TakaraBio). As a negative control, a synthetic peptide containing 6xHis and C-tag was synthesized (Sigma-Aldrich).

Antigens (0.2 μg per 100 μL per well) were pre-coated on Nunc Immuno Module plates in carbonate-bicarbonate buffer (Sigma) at 4 °C overnight. Washing and blocking were performed three times using 300 μL of SuperBlock-T20 (Thermo Fisher Scientific). The concentrations of purified sdAbs were measured using a Qubit Protein Assay kit (Life Technologies). Primary antibodies (10 μg/mL) premixed with an anti-C-tag biotin conjugate (1:1000) were incubated at 4 °C overnight. For background measurement, the anti-C-tag biotin conjugate alone was used. After washing five times with 300 μL of PBS-T (0.1 M sodium phosphate, 0.15 M NaCl, 0.02% Tween20, pH 7.2), plates were re-blocked with 300 μL of SuperBlock-T20, and incubated with streptavidin-HRP (1:10000) at RT for 1 h. The plates were then washed three times with PBS-T and twice with PBS. After chromogen was applied, absorbance values were measured at a wavelength of 620 nm.

### Autoreactivity profiling

Recombinant sdAbs were mixed with CaptureSelect anti-C-tag biotin conjugate, and sent to Cambridge Protein Arrays Ltd. for HuProt v2 Proteome Microarray analysis. This array provides the largest number of individually purified human proteins, representing more than 15,000 genes that cover ~75% of the annotated human genome. Briefly, reaction was performed as follows. Arrays were blocked; samples were diluted with blocking buffer to a final concentration of 7.5 μg/mL sdAbs plus 2.5 μg/mL anti-C-tag biotin conjugate. Arrays were probed with diluted samples, washed three times with PBS-T, probed with Streptavidin-532 (2.0 μg/mL in blocking buffer), washed three times with PBS-T, and rinsed with DW. Finally, the arrays were scanned on a Tecan LS400 microarray scanner at an excitation wavelength of 532 nm. Briefly, data analysis was carried out as follows. Negative control data lacking primary probe were subtracted. Fluorescence signal intensities were averaged for duplicate spots. Z-score was calculated as:


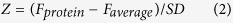


where *F*_*protein*_ is the fluorescence signal intensity of a specific protein, *F*_*average*_ is the mean fluorescence signal intensity of all protein spots on the array (excluding control spots), and *SD* denotes the standard deviation of all protein spots on the array (excluding control spots). Proteins were initially filtered on the basis of the threshold of Z-score >3.5, signal-to-noise ratio >2.5, and relative standard deviation of duplicate spots <0.35. Results were manually confirmed, and interactions at Z-score below 3.5 were included when visually validated.

### Clonotype diversity analysis

CDR-H3 sequences with exactly the same nucleotide sequences and VJ pairing were considered a single clonotype. Rarefaction analysis for estimation of clonotype diversity was performed using the iNEXT package. Selection pressure was quantified using the BASELINe web tool and R source code^46^ (http://selection.med.yale.edu/baseline/). Sigma estimators from the focused test method were used for visualization purposes.

### AAIndexScore

The AAIndex (http://www.genome.jp/aaindex/) dataset was retrieved from the BioSeqClass package. We selected 78 AAIndices whose descriptions contain the following terms; “hydro”, “charge”, “polar”, “flexi”, and “distribution”. The net value of each AAIndex was calculated for each CDR-H3 sequence for which the ratio of two iTRAQ reporters was determined. Principal component analysis (PCA) and clustering analysis were conducted primarily using the FactoMineR package. We utilized the ratios of the iTRAQ intensities as a surrogate indicator of the “propensity” of condensation to cryoprecipitate. The rescaled iTRAQ ratio was designated as “CryoglobulinIndex”. Multiple regression analysis showed that a composite score derived from PC1 and PC2 modestly correlates to CryoglobulinIndex. Hierarchical clustering splits the AAIndices into three clusters scattered on the PC1-PC2 coordinate plane, indicating that at most three parameters should be sufficient for constructing a prediction model for CryoglobulinIndex. Regression with too many variables would lead to overfitting and multicollinearity issues, and thus should be avoided. Multiple regression analysis was conducted by the lm function implemented in R. All triple combinations of selected AAIndices were tested to find the best combination. When the maximum variance inflation factor (VIF) exceeds 3.0, that model was discarded. CDR-H3 sequences with CryoglobulinIndex of 0.6 or higher were defined as enriched in cryoprecipitate. Based on this definition, receiver operating characteristic (ROC) analysis was performed using the roc function implemented in the pROC package, and the area under the curve (AUC) was calculated. A triple combination of AAIndices resulting in the highest AUC was selected, and the regression score was designated as “AAIndexScore” (Supplementary Table 3).

### Clonotype dynamics analysis

Based on the relative weight on AAIndexScore, amino acid residues were categorized by a hierarchical clustering approach. A small alphabetical character was assigned to each of the categories. The distance between a pair of CDR-H3 sequences was defined as the edit distance of two CDR sequences after each residue was replaced with the corresponding category character. Edit distance was calculated using EditDistance, a built-in function in Mathematica. The distance matrix was converted to a coordinate matrix via principal coordinate analysis (PCoA) using the cmdscale function implemented in the stats package. The coordinate matrix was then used for linear discriminant analysis using the lda function in the MASS package. Next, an amino-acid-level similarity network was constructed in which each CDR-H3 was connected to their nearest counterparts based on the above-mentioned distance scheme. The resultant unidirectional network reflects amino-acid-level similarity in the context of HCV-CG, and CDR-H3 sequences sharing similar motifs are more likely to be densely connected. Note that this network, in principle, does not contain nucleotide-level information reflecting the ontogeny and evolution of each CDR-H3 clonotype; rather, phenotypic similarities can be dissected at the level of amino acid sequences. The network analysis was performed using the igraph package. Five functions were tested for community identification: edge.betweenness.community, walktrap.community, fastgreedy.community, leading.eigenvector.community, and multilevel.community. The fast-greedy and multilevel optimization algorisms classified UT1.1-1.3 sequences in a single community. Similar analysis using Mathematica’s built-in function FindGraphCommunity showed similar results with the default settings. On the basis of abovementioned observations, we concluded that there is a single distinct community, i.e. sub-repertoire, containing UT1.1-1.3 sequences, and we decided to use the result of multilevel optimization algorism as representative.

### Statistical analysis

This study is a single-patient report. No power calculation was performed to predetermine the sample size. No inclusion/exclusion criteria or randomization procedures were applied. The investigator was not blinded throughout the study, except the HuProt protein array experiment for screening candidate autoantigens. Experiments using clinical specimen were performed without duplicates unless otherwise stated. The statistical test used and significance code are as specified in the main text and/or the legend of each figure. When parametric tests were applied, equality of variances was validated by the F-test. When multiple groups were compared, *P* values were adjusted for multiple comparisons. All statistical tests were conducted in a two-sided manner. Data are presented as the mean ± s.e.m. unless otherwise specified. Statistical analysis was performed using R.

## Additional Information

****Accession codes:**** The V_H_ repertoire sequencing datasets have been deposited in the DDBJ Sequence Read Archive (SRA) under the accession number DRA004494. The mass spectrometry dataset has been deposited in the ProteomeXchange Consortium under the accession number PXD002475. The coding sequences in the expression vectors of UT1.1, UT1.2, and UT1.3 have been deposited in GenBank under the accession numbers LC128802, LC128803, and LC128804, respectively.

**How to cite this article**: Ogishi, M. *et al.* Delineation of autoantibody repertoire through differential proteogenomics in hepatitis C virus-induced cryoglobulinemia. *Sci. Rep.* **6**, 29532; doi: 10.1038/srep29532 (2016).

## Supplementary Material

Supplementary Information

Supplementary Dataset 1

Supplementary Dataset 2

Supplementary Dataset 3

Supplementary Dataset 4

Supplementary Dataset 5
