# Supplementary material for: Delineation of autoantibody repertoire through differential proteogenomics in hepatitis C virus-induced cryoglobulinemia
Source: Sci Rep. 2016 Jul 12;6:29532. doi: 10.1038/srep29532 (PMC4941579; doi:10.1038/srep29532)
Supplement: Supplementary Information [file srep29532-s1.pdf]

# Supplementary Figures and Tables for

# Delineation of autoantibody repertoire through differential proteogenomics in hepatitis C virus-induced cryoglobulinemia

Masato Ogishi, Hiroshi Yotsuyanagi, Kyoji Moriya, and Kazuhiko Koike

**Supplementary Figures**

a

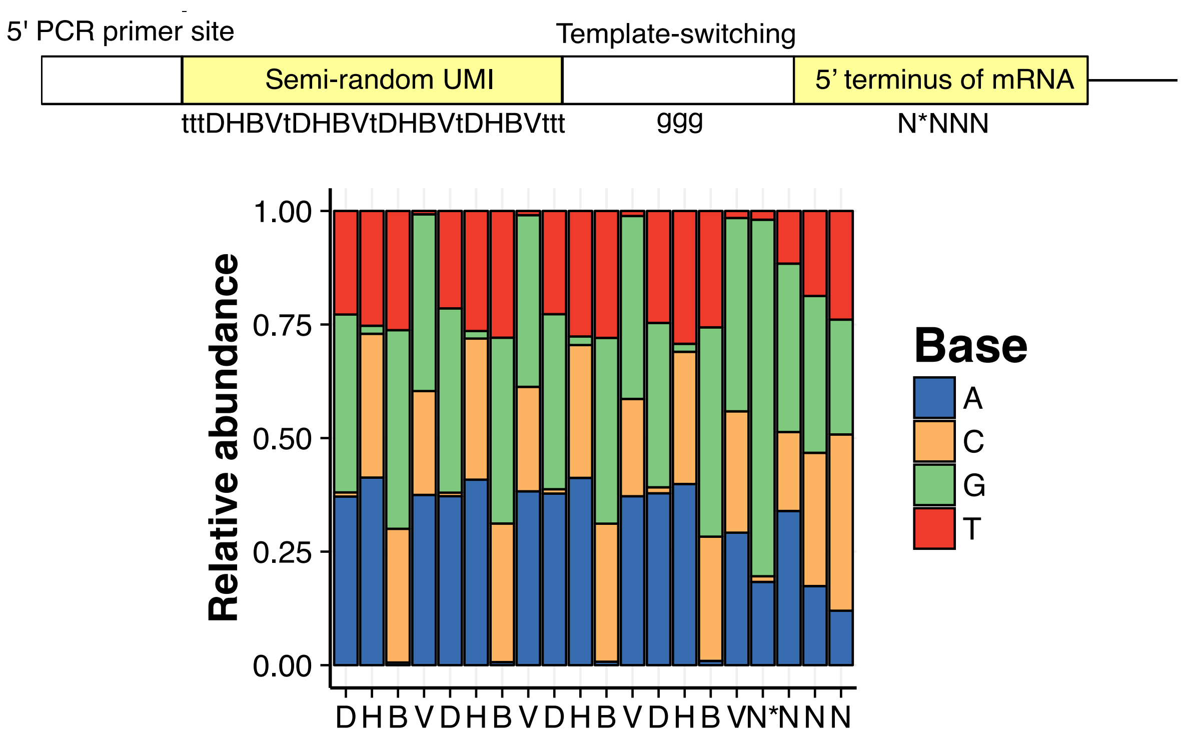


b

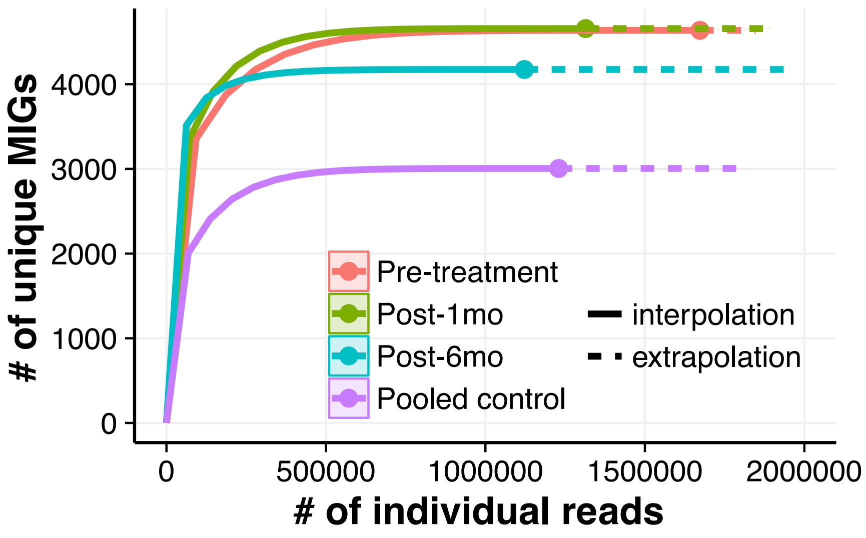

**Supplementary Figure 1.** Antibody repertoire sequencing. (**a**) Top: structure of unique molecular identifier (UMI) barcode. Bottom: relative abundance of bases observed in each position of the barcode. The barcode sequence was incorporated in the template-switching oligonucleotide. The low frequency of unintended bases at each position of semi-random UMI (<1%) validates the accuracy of the whole process of barcode synthesis, deep sequencing, and UMI alignment and assembly using MiGEC pipeline. The obelisk mark denotes the 5'-terminus of mRNA. The preferential use of G owing to template-switching reaction was observed. The four 5'-terminal bases were also included in the MiGEC de-multiplexing process to distinguish, if any, two sequences having exactly the same UMI sequence. (**b**) Rarefaction analysis of unique molecular identifier groups (MIGs) observed. Dots represent the total number of individual reads in each deep sequencing data. The saturating pattern of the curves indicates that adequate sequence depth was achieved to thoroughly detect the cDNA molecules tagged with unique UMI. Approximately two to three thousand unique MIGs were identified in each of the samples.

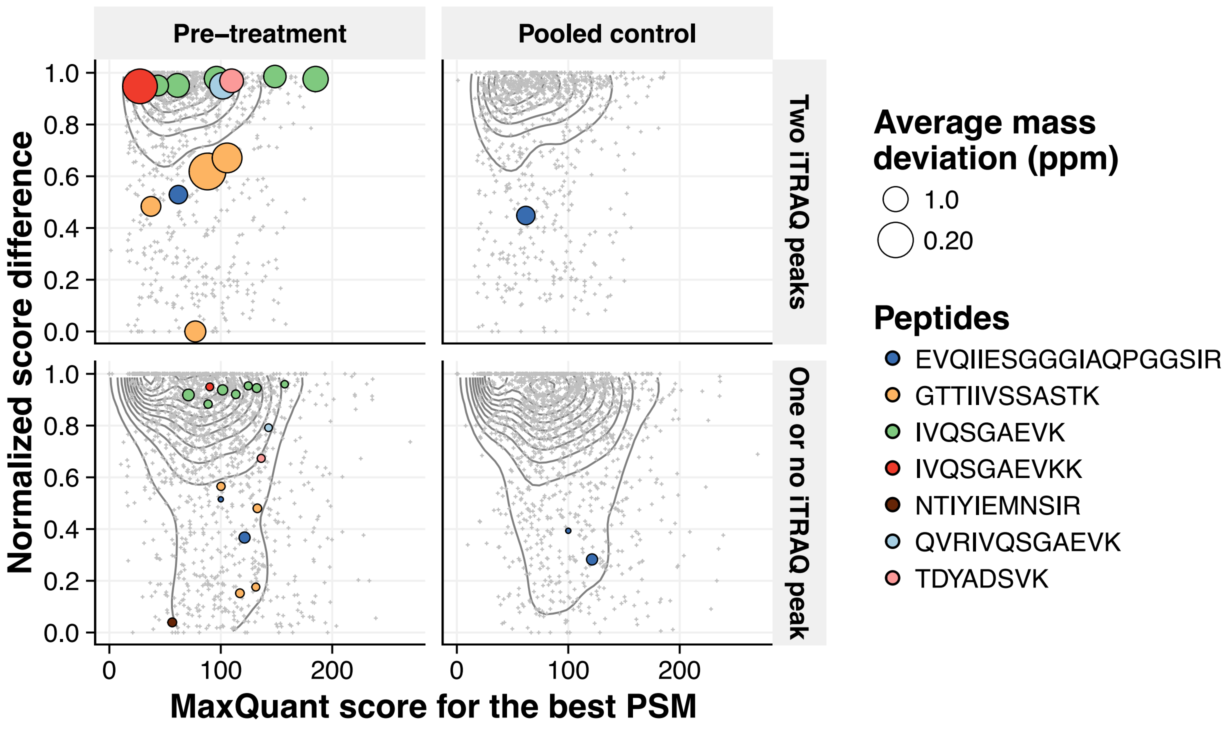


**Supplementary Figure 2.** Peptide-spectrum matches (PSMs). Isobarically tagged mass spectra were matched against the in-house $V_H$ sequence database constructed by deep sequencing using MaxQuant software. PSMs were split on the basis of whether their accompanying iTRAQ peaks were successfully detected. PSMs with two iTRAQ peaks successfully identified generally have larger difference between the top-ranked and second-ranked PSM scores, indicating unambiguous matching. The colored dots represent the evidence PSMs for the representative $V_H$ sequences identified as the most enriched ones in cryoprecipitate (Fig. 1e). The size represents the average mass deviation, which has been shown as a good indicator for distinguishing correctly matched PSMs from inaccurately matched ones. Evidence PSMs with two iTRAQ peaks have lower average mass deviations, indicating more accurate matching. The right panel shows the same matching experiment using $V_H$ database of pooled control. None but EVQIIESGGGIAQPGGSIR peptides were detected, suggesting their uniqueness in the pre-treatment $V_H$ database. From these observations, PSMs without two iTRAQ peaks were discarded in subsequent analyses.

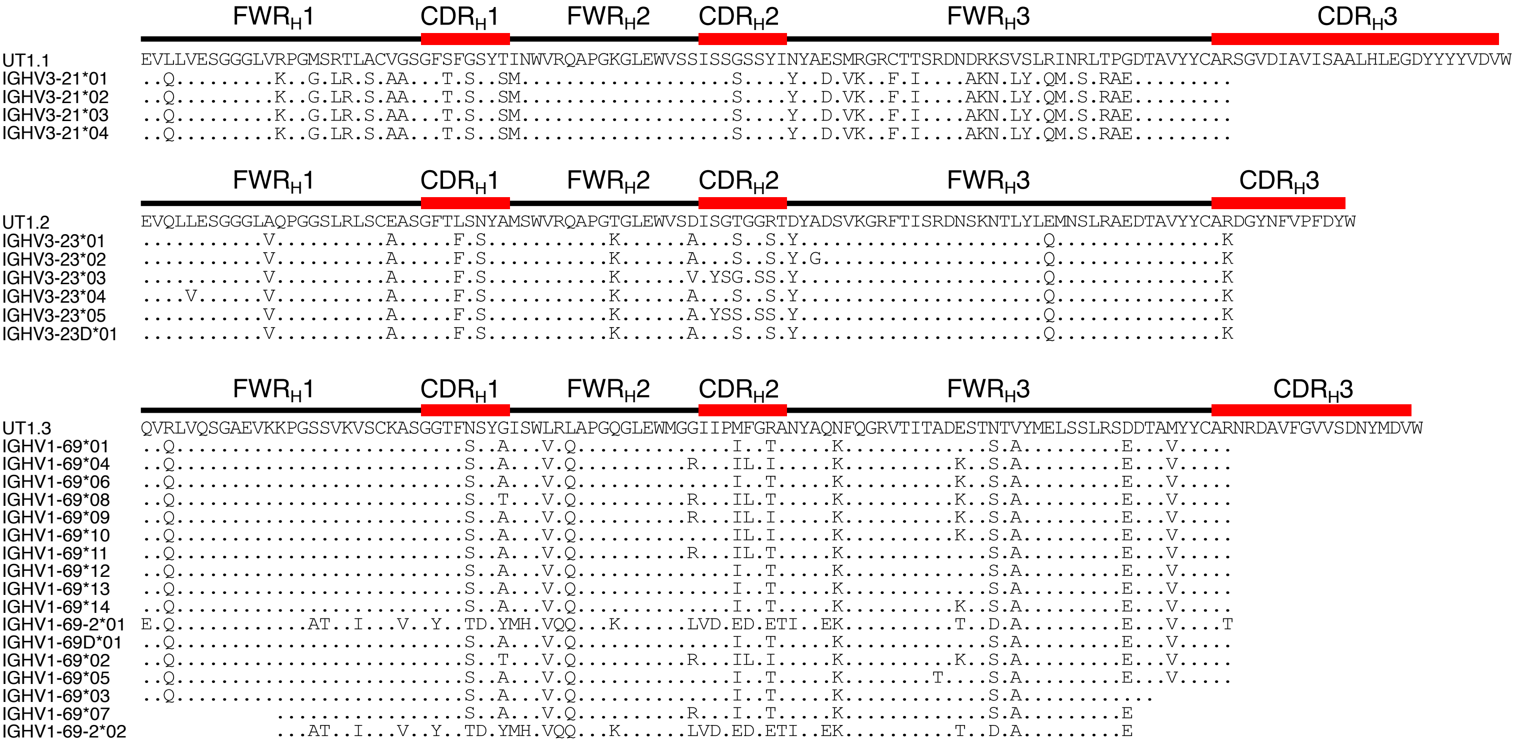


**Supplementary Figure 3.** Complete sequence alignments of three proteogenomically identified representative $V_H$ sequences. Notably, high rates of amino acid substitutions were observed not only in the complementarity-determining regions (CDRs) but also in the framework regions (FWRs), indicating a substantial positive selection pressure over these $V_H$ sequences.

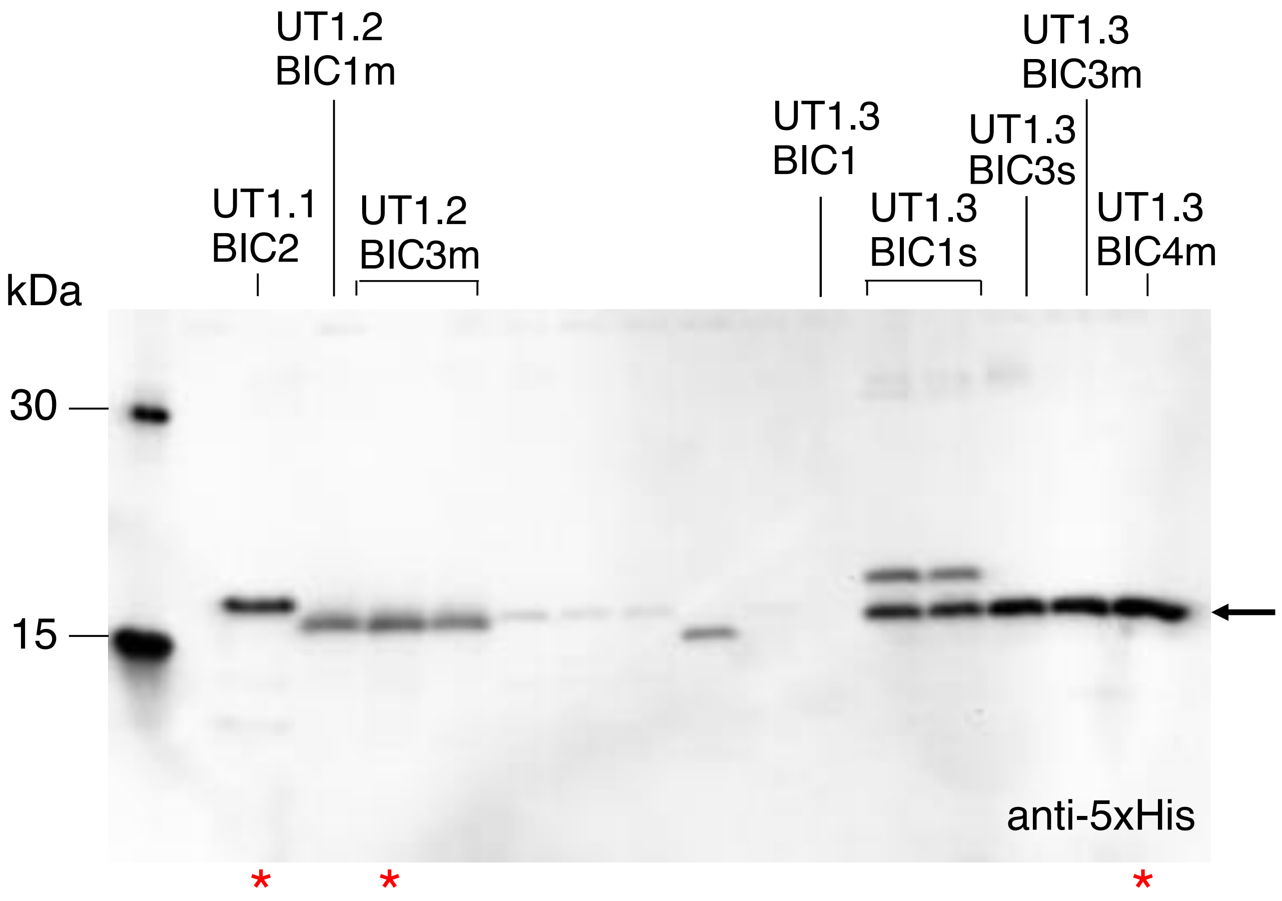


**Supplementary Figure 4.** Expression of representative $V_H$ sequences as single-domain antibodies (sdAbs). Proteins were expressed using the *Brevibacillus* expression system. Red marks indicate the clones used in subsequent reactivity profiling experiments (Fig. 2).

a

b

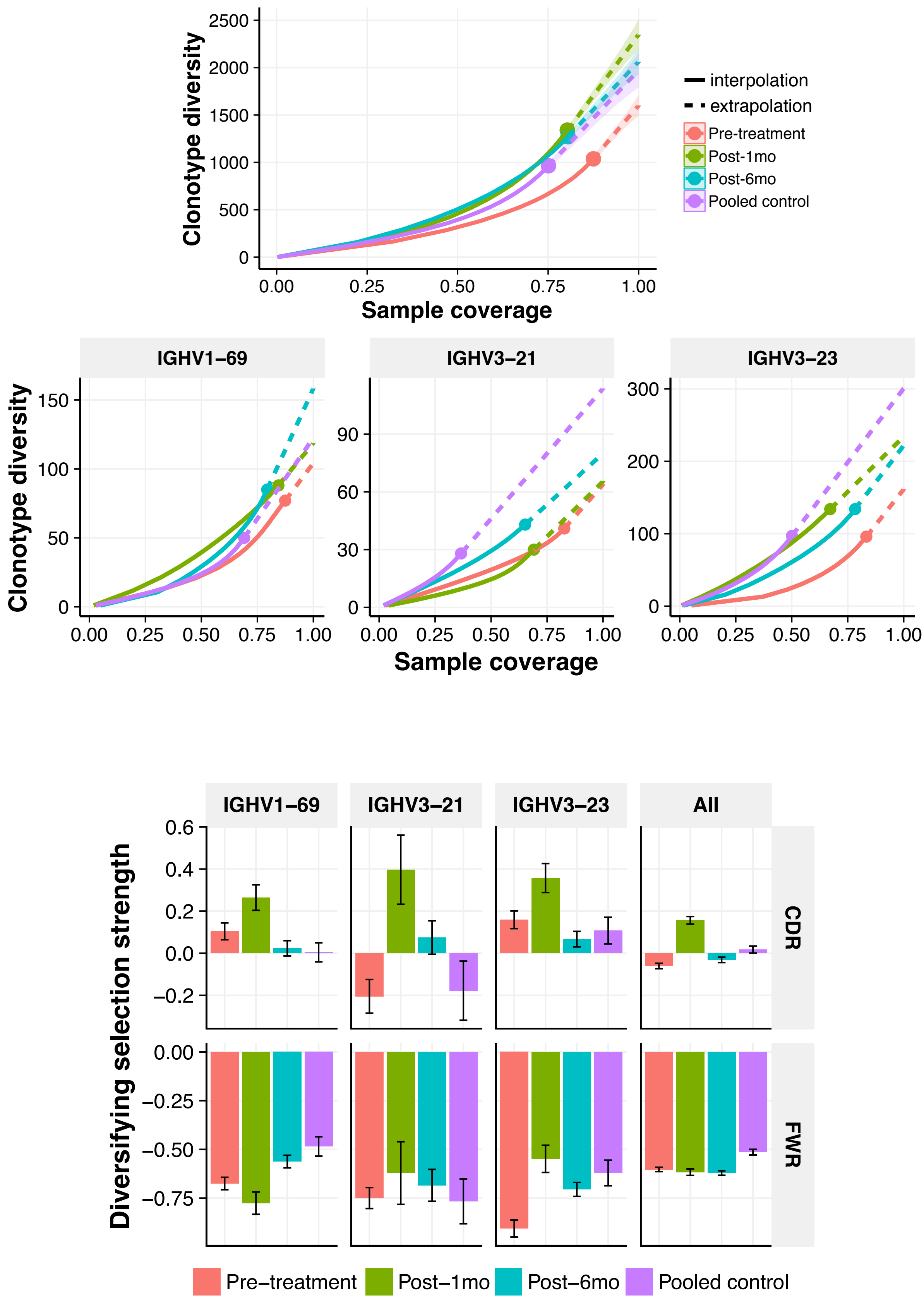

a
Clonotype diversity
Sample coverage
interpolation
extrapolation
Pre-treatment
Post-1mo
Post-6mo
Pooled control
IGHV1–69
IGHV3–21
IGHV3–23
b
Diversifying selection strength
IGHV1–69
IGHV3–21
IGHV3–23
All
CDR
FWR
Pre–treatment
Post–1mo
Post–6mo
Pooled control

**Supplementary Figure 5.** Re-diversification of CDR-H3 repertoires after antiviral therapy. (**a**) Rarefaction analysis of clonotype diversity. Top: whole repertoire. The lines and ribbons indicate means and 95% confidence intervals estimated by bootstrapping (n = 1000). Bottom: IGHV1-69, IGHV3-21 and IGHV3-23 sub-repertoires. Clonotype diversity increased to the level of the pooled control after the initiation of antiviral therapy. The lines indicate means estimated by bootstrapping (n = 1000). (**b**) Diversifying selection pressure analysis using the BASELINe program (http://selection.med.yale.edu/baseline/). The principle is based on the relative abundance of nonsynonymous mutations to synonymous ones. The focused sigma estimator calculated for each $V_H$ sequence was used for visualization. A positive value and a negative value indicate diversification and conservation at the level of amino acids, respectively. In CDR, a strong diversifying trend was observed at 1 month, indicating that the overwhelmingly driving antigen, i.e., HCV, was eradicated owing to antiviral therapy. At six months, selection pressure returned to the neutral level. On the other hand, in FDR, conservative selections were consistently observed. CDR, complementarity-determining region. FWR, framework region. Bars indicate mean ± s.e.m.

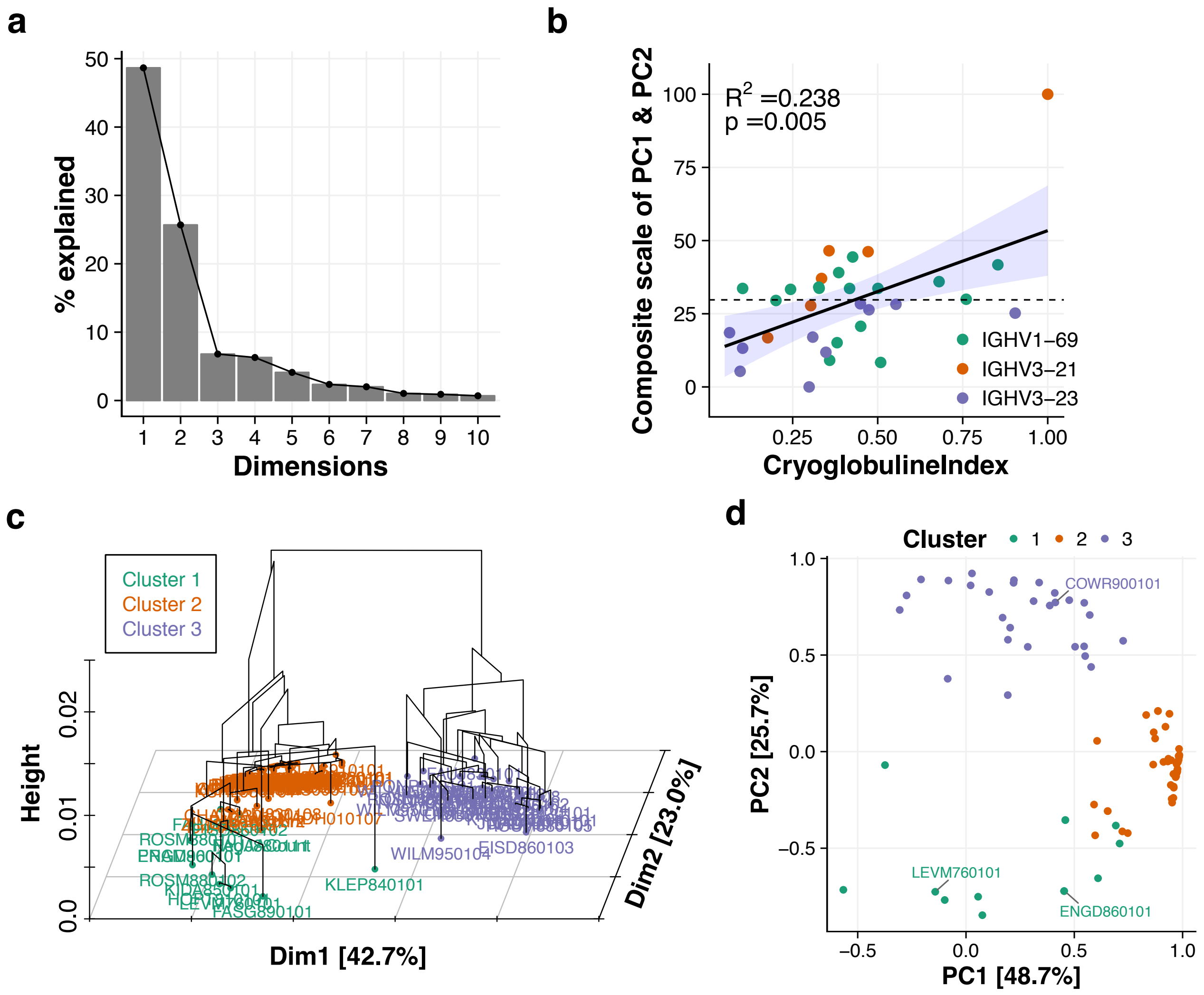


**Supplementary Figure 6.** Parametrization of the propensity of cryoprecipitation with AAIndex. The biochemical properties of the amino acids of CDR-H3 were used in the exploration of the best axis that correlates with CryoglobulineIndex, which is defined as a rescaled ratio of iTRAQ signal intensities (Fig. 1d). Seventy-eight AAIndices were selected for model construction. For each CDR-H3 sequence, the net value of each AAIndex was calculated. (**a**) A principal component analysis (PCA) revealed that two dimensions, PC1 and PC2, explain nearly 75% of the variance of the whole dataset. (**b**) Multiple regression analyses of PC1 and PC2 indicated that a composite score of these two dimensions showed a considerable correlation to CryoglobulineIndex. A dash line indicates the best threshold value determined by receiver operating characteristic (ROC) analysis. This result convincingly supports the hypothesis that a substantial fraction of the condensation of a certain antibody to cryoprecipitate is determined solely by the biochemical properties of its CDR-H3. (**c, d**) Hierarchical clustering of AAIndices resulted in three clusters. This led us to the ideation that a maximum of three AAIndices should be sufficient for explaining the CryoglobulineIndex. In (**d**), three AAIndices used in AAIndexScore (Fig. 3a, b and Supplementary Table 3) were labeled.

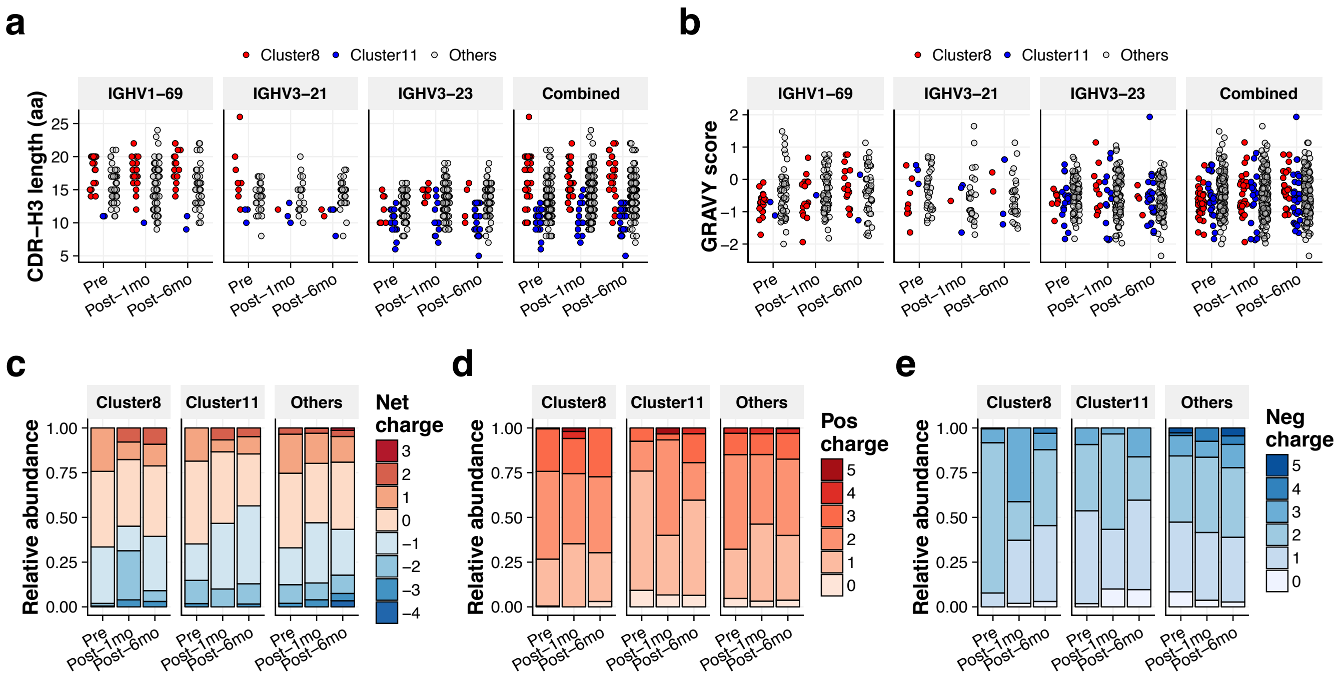


**Supplementary Figure 7.** General features of CDR-H3 repertoires. (**a**) CDR-H3 length. CDR-H3 sequences in Cluster8 were longer than those in Cluster11. For the definition of the clusters, see the main text and the legend of Figure 3d. (**b**) GRAVY score. The scores did not significantly differ between the clusters. No differences were observed between time points. (**c-e**) Charges. CDR-H3 sequences with more negatively charged residues were abundant in Cluster8 at pre-treatment, but the difference disappeared after six months. This observation might reflect the dominance of negatively charged antibodies against positively charged HCV virions. No significant differences were observed between time points in positive and net charges.

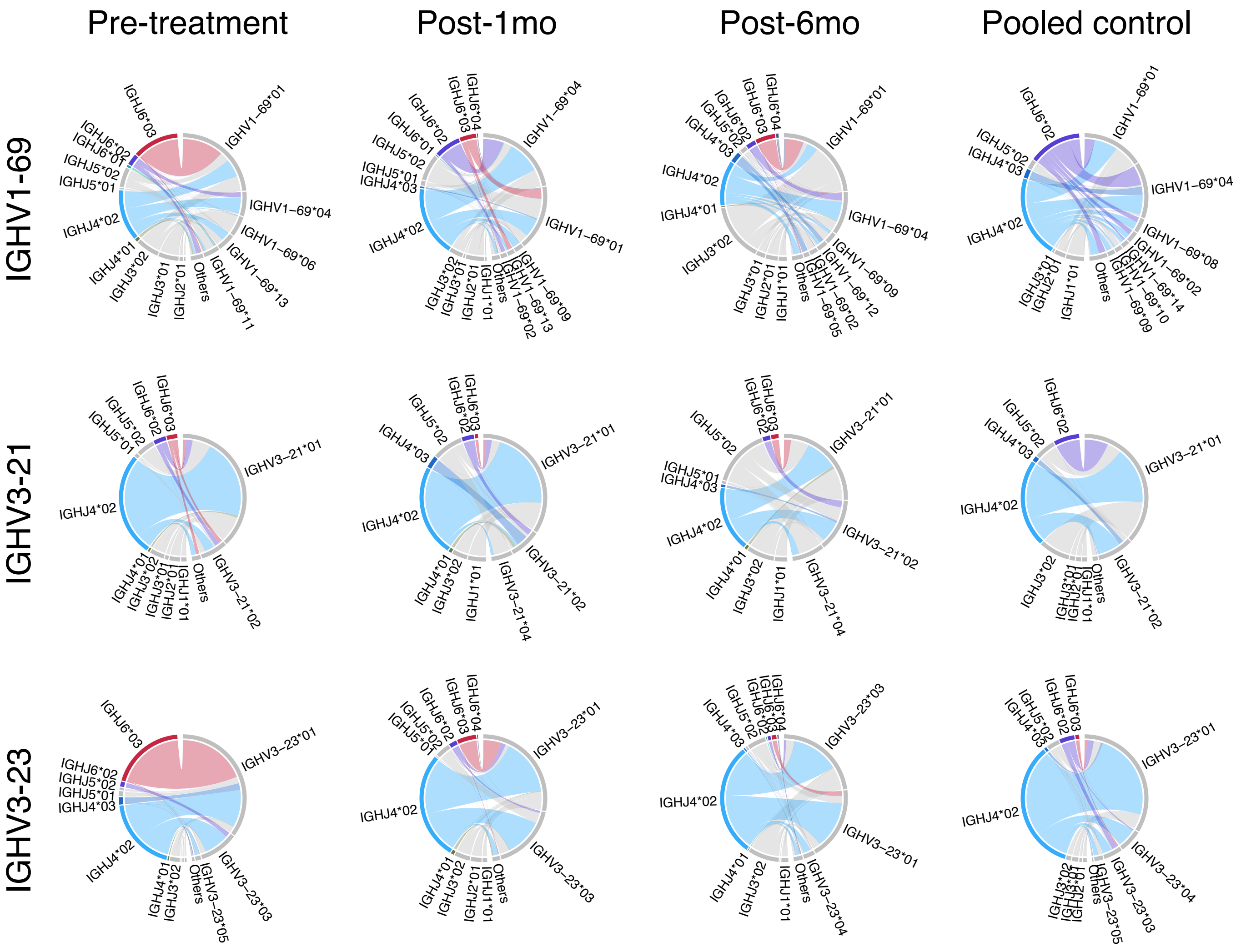


**Supplementary Figure 8.** Longitudinal dynamics of V-J segment usage in the CDR-H3 repertoires over antiviral therapy. A striking expansion of CDR-H3 sequences derived from IGHJ6*03 was noted in pre-treatment repertoires. In particular, a single thick band linking IGHJ6*03 and a small subset of IGHV gene segments was noticeable in IGHV1-69 and IGHV3-23 repertoires, suggesting the expansion of highly similar antibodies with overlapping V-J segment usage. In contrast, CDR-H3 sequences of IGHJ6*03 origin diminished in a time-dependent manner after antiviral therapy. In contrast, almost no IGHJ6*03 was detected in the pooled control repertoires.

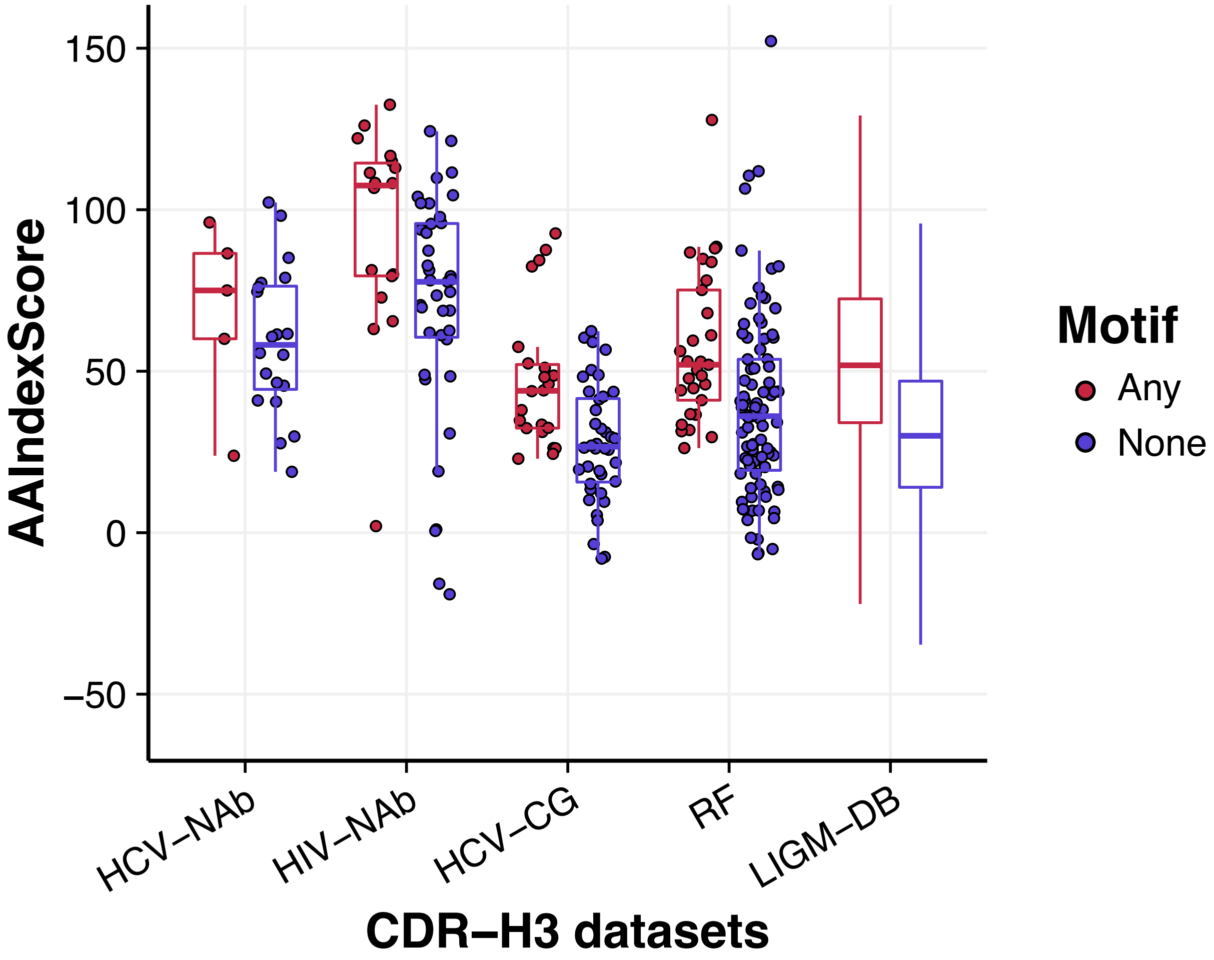
150
100
50
0
−50
AAIndexScore
HCV−NAb
HIV−NAb
HCV−CG
RF
LIGM−DB
CDR−H3 datasets
Motif
Any
None

**Supplementary Figure 9.** External applicability of AAIndexScore to publicly available CDR-H3 sequence datasets. The source of the datasets were as follows: HCV-NAb, manually corrected CDR-H3 sequences of human anti-HCV E2 antibodies reviewed elsewhere (Supplementary Table 4); HIV-Nab, the dataset downloaded from the bNAber database (as of 2014/01/27) (Supplementary Table 5); HCV-CG, the human CDR-H3 sequences of peripheral blood mononuclear cells isolated from patients with HCV-induced cryoglobulinemia. Sequence data were retrieved from IMGT/LIGM-DB. Original research articles were manually inspected, and sequences derived from other sources (e.g., healthy volunteers, patients infected with HCV but CG(-)) were discarded (Supplementary Table 6); RF, the human CDR-H3 sequences retrieved from GenBank with the query "rheumatoid factor" (Supplementary Table 7); LIGM-DB, the whole dataset of human CDR-H3 sequences downloaded from IMGT/LIGM-DB (as of 2015/09/06) (Supplementary Table 8). CDR-H3 regions were identified using IMGT/HighV-Quest (http://www.imgt.org/HighV-QUEST/) whenever full $V_H$ sequences were available. In the plot, sequences were split by the presence or absence of the motifs discussed in Figure 4. HCV-NAbs and HIV-NAbs showed significantly higher AAIndexScores than other datasets ($P < 0.001$). Although not statistically significant, motif-containing NAbs appeared to show even higher AAIndexScores. Multiple groups were compared by a Wilcoxon rank sum test using a pairwise.wilcox.test function. *P* values were adjusted by the Benjamini and Yekutieli method, which is a false discovery rate (FDR)-controlling procedure providing provide less stringent control of Type I errors.

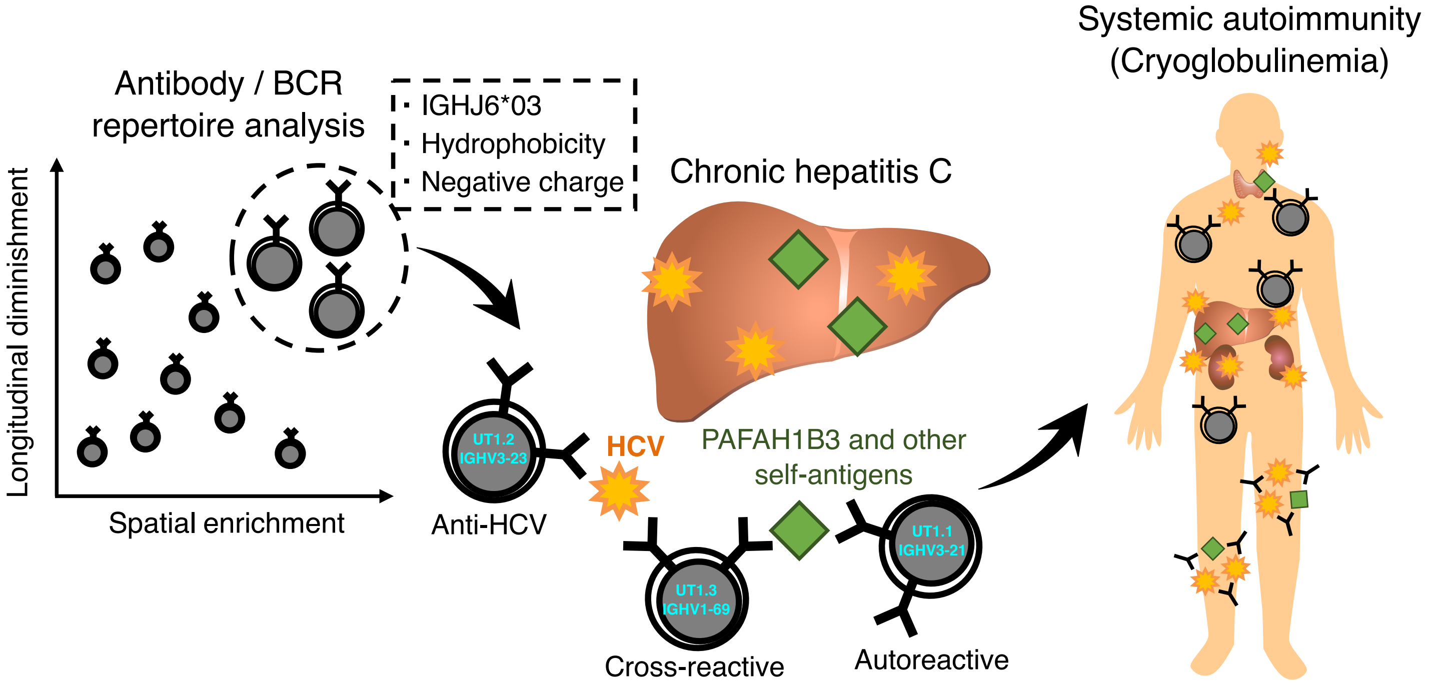


**Supplementary Figure 10.** Schematic summary of the findings and possible pathogenic mechanisms of HCV-induced cryoglobulinemia (HCV-CG). Left: we focused on the antibody repertoire that (i) was highly enriched in a disease-specific compartment, i.e., cryoprecipitate, and (ii) decreased in relative abundance after eradication of HCV by direct-acting antiviral treatment. Computational analyses revealed a distinct CDR-H3 sub-repertoire, in which IGHJ6*03-derived, long, hydrophobic, negatively charged CDR-H3 sequences were particularly enriched. BCR, B cell receptor. Middle: characterization of three representative antibodies suggested the presence of anti-HCV antibodies, autoreactive antibodies, and cross-reactive antibodies in cryoprecipitate. Possibly, B cells infiltrating in the liver may be sensitized from both HCV and self-antigens discharged from destroyed hepatocytes, differentiating into plasma cells. Right: subsequent systemic employment of those cross-reactive plasma cells may cause the formation of cryoprecipitate and some of the extrahepatic manifestations of chronic hepatitis C. Illustrations were modified from the resources distributed in the Togo picture gallery (http://g86.dbcls.jp/~togoriv/), licensed under CC-BY 4.0 ©Togo picture gallery by the Database Center for Life Science (DBCLS), Japan.

# Supplementary Tables

**Supplementary Table 1.** Clinical characteristics of the patient studied. The patient (UT1) was a 70 year-old female chronically infected with HCV genotype 1b. The source of her infection was not apparent. Her cryoglobulin was type III, as serum monoclonal protein was not detected by immunoelectrophoresis. She was treated with a combined regimen of daclatasvir (DCV) and asunaprevir (ASV). She achieved SVR12, and her viremia was kept undetectable thereafter. n.a., not available.

| Date | Day0 | Day28(4w) | Day84(12w) | Day112(16w) | Day168(24w) | Day266(38w) | Day368(52w) |
|---|---|---|---|---|---|---|---|
| DAA | DCV60 + ASV200 | | | DCV60 + ASV100 | | None | |
| T-Bil [mg/dL] | n.a. | n.a. | 1.3 | 1.1 | n.a. | 1.0 | 0.9 |
| AST [IU/L] | 20 | 13 | 65† | 22 | 18 | 18 | 19 |
| ALT [IU/L] | 16 | 10 | 114† | 19 | 11 | 12 | 14 |
| ALP [IU/L] | 262 | 316 | n.a. | 342 | 289 | 282 | 299 |
| γGTP [IU/L] | 17 | 15 | 16 | 23 | n.a. | 14 | 15 |
| Alb [g/dL] | 3.7 | 3.6 | 3.8 | 3.8 | 3.7 | 3.8 | n.a. |
| Plt [x10E4/μL] | 28.5 | 29.4 | 24.4 | 24.6 | 24.8 | 25.1 | n.a. |
| RF [U/mL] | 82 | 77 | 66 | 65 | 59 | 47 | 44 |
| C3 [mg/dL] | n.a. | n.a. | n.a. | n.a. | n.a. | n.a. | 121 |
| C4 [mg/dL] | n.a. | n.a. | n.a. | n.a. | n.a. | n.a. | 21 |
| IgM [mg/dL] | 63 | 62 | 65 | 91 | 77 | 73 | 68 |
| IgG [mg/dL] | 2190 | 2185 | 2287 | 2354 | 2514 | 2181 | 2223 |
| HCV Viral Load [LogIU/mL] | 5.4 | <1.2 | undetectable | undetectable | undetectable | undetectable | undetectable |

†: A slight increase of AST and ALT was noted. ASV-induced liver injury was suspected, and the dose of ASV was decreased down to 100mg.

**Supplementary Table 2.** Oligonucleotides used in this study. On the basis of previous study, we utilized locked nucleic acid (LNA) at the 3' terminus. TSO, a template-switching oligo. RT, A reverse-transcription primer specific to IgG constant region with adapter sequence for 3'-RACE. HC, heal-carrier oligo for step-out suppression PCR. HS, heal-specific primer for step-out suppression PCR; Either N, NN, or NNN was attached to the primers to prevent signal saturation through the base calling process in MiSeq.

| Name | Sequence |
|---|---|
| IgSeq-TSO_v3 | ACAGCAGGTCAGTCAAGCAGTATTTDHBVTDHBVTDHBVTDHBVTTTgg**G**[†] |
| IgSeq-RT-IGHV_v3 | AGCAGTAGCAGCAGTTCGATAAGGTGTGCACGCCGCTGGTC |
| IgSeq-F-HC_v3 | CGGATAACAATTTCACACAGGGCACAGCAGGTCAGTCAAGCAGTA |
| IgSeq-F-HS-N1_v3 | NCGGATAACAATTTCACACAGGGC |
| IgSeq-F-HS-N2_v3 | NNCGGATAACAATTTCACACAGGGC |
| IgSeq-F-HS-N3_v3 | NNNCGGATAACAATTTCACACAGGGC |
| IgSeq-R1_v3 | AGCAGTAGCAGCAGTTCGATAA |
| IgSeq-R2-N1-IGHV_v3 | NACCGATGGGCCCTTGGTG |
| IgSeq-R2-N2-IGHV_v3 | NNACCGATGGGCCCTTGGTG |
| IgSeq-R2-N3-IGHV_v3 | NNNACCGATGGGCCCTTGGTG |

[†]: Template-switching oligo with semi-random unique molecular identifier (UMI) containing the following bases: g , ribosyl guanine; **G**, LNA guanine.

**Supplementary Table 3.** Construction of AAIndexScore. Best triple combination of AAIndices resulting in the highest area under the curve (AUC) value for distinguishing CDR-H3 sequences highly enriched in cryoprecipitate from non-enriched ones. The resultant multiple regression score was designated as "AAIndexScore" (Fig. 3a, b). The three selected AAIndices were classified into Clusters 1 and 3 in the principal component analysis, indicating that PC2 represented the most predictive axis for CryoglobulinIndex (Supplementary Fig. 6). PCA, principal component analysis. MRA, multiple regression analysis. VIF, variance inflation factor. MRA coefficient estimators are presented as mean ± s.e.m.

| AAIndex | | PCA and clustering | | | MRA | | |
|---|---|---|---|---|---|---|---|
| Name | Description | PC1 | PC2 | Cluster | Estimator | *P* value | VIF |
| COWR900101 | Hydrophobicity index, 3.0 pH (Cowan-Whittaker, 1990) | 0.41 | 0.77 | 3 | 1.07 ± 0.37 | 0.007 | 1.45 |
| ENGD860101 | Hydrophobicity index (Engelman et al., 1986) | 0.45 | -0.72 | 1 | 0.16 ± 0.07 | 0.034 | 1.58 |
| LEVM760101 | Hydrophobic parameter (Levitt, 1976) | -0.14 | -0.73 | 1 | -0.43 ± 0.26 | 0.105 | 2.11 |
